# Ethiopia's Potato Seed System: Regulatory Challenges, Quality Assurance Issues, and Pathways for Improvement ─ A mini review

**DOI:** 10.12688/f1000research.158093.2

**Published:** 2025-02-10

**Authors:** Lemma Tessema, Ebrahim Seid

**Affiliations:** 1 Ethiopian Institute of Agricultural Research, Holetta Agricultural Research Centre, P.O.Box 2003, Addis Ababa, Ethiopia

**Keywords:** Potato seed system, quality assurance, regulatory framework, food security

## Abstract

**Background:**

Ethiopia’s potato seed system is currently hindered by a disjointed regulatory framework, insufficient quality assurance processes, and a lack of collaboration among stakeholders, which collectively impede agricultural productivity and food security. The regulatory environment is characterized by inconsistent seed certification practices and a dependence on informal seed sources, negatively affecting the quality of potato seeds available to farmers. Although potatoes play a crucial role in global food security by providing high yields compared to other staple crops, the sector grapples with significant challenges due to bureaucratic inefficiencies, limited technical expertise, and the widespread presence of counterfeit seeds throughout the value chain.

**Methods:**

To tackle these challenges, this mini-review outlines potential improvements, stressing the necessity for a unified regulatory framework tailored to the unique issues of vegetatively propagated crops. For this reason, we have used different literature source from web of science, Pubmed, Google Scholar, ResearchGate and other scientific websites. We have documented the most relevant information focusing on potato seed system, regulatory frameworks, quality assurance bottlenecks and pathways for improvement across the seed value chain.

**Results:**

our paper highlights key recommendations that includes enhancing training for regulatory staff, investing in research for disease-resistant varieties, and bolstering public-private partnerships to encourage innovation and resource sharing. Furthermore, improving traceability in the seed supply chain is essential for maintaining seed quality and integrity.

**Conclusions:**

This review calls for a comprehensive strategy that fosters stakeholder engagement and promotes sustainable practices to rejuvenate Ethiopia’s potato seed system, thereby supporting the livelihoods of millions of smallholder farmers and strengthening national food security through integrated seed sector development and capacity improvement of the seed regulatory body as well as smallholder farmers.

## Introduction

The potato ( *Solanum tuberosum* L.), often referred to as the “perfect food” or the “underground apple,” is essential for global food security and nutrition. As the third most important food crop, it is poised to become a key player in the global food security system, particularly as the yields of other cereal crops approach their limits ( Devaux et al., 2021; Wijesinha-Bettoni and Mouille, 2019). The global average yield gains from adapting to climate change range between 10 and 17% according to various climate models. Compared to other major crops, potato farming produces fewer greenhouse gas emissions, making it a climate-smart choice due to the anticipated increases in yield ( Jennings et al., 2020).

The potato significantly supports global food security, nutrition, and healthy diets for millions of households and for creating diversified agri-food systems ( Devaux et al., 2021; Wijesinha-Bettoni and Mouille, 2019). In many developing countries, including Ethiopia, where rapid population growth, shrinking arable land, and climate change are critical challenges to food security, potatoes emerge as a strategic crop. Potatoes are particularly valuable because they yield more food per unit of land compared to other staple crops ( Mijena et al., 2022; Devaux et al., 2021).

Ethiopia’s potato seed system faces critical challenges that impede agricultural productivity and food security ( Hirpa et al., 2010). The regulatory framework governing seed production and distribution is fragmented and often ineffective, leading to a lack of clarity and consistency in seed certification and quality assurance processes ( Getnet et al., 2023; Hirpa et al., 2010; Tessema et al., 2023a). This regulatory uncertainty discourages investment and participation from local farmers and seed producers, exacerbating the reliance on informal seed sources that compromise crop quality ( Schulz et al., 2013; Katrin et al., 2023). Furthermore, quality assurance mechanisms are insufficient, resulting in the proliferation of low-quality seeds that are vulnerable to diseases and pests ( Alemu et al., 2023; Nigussie et al., 2020; Tessema et al., 2023b). These issues collectively hinder the potential of the potato sector, which is vital for both the economy and nutrition in Ethiopia ( Getnet et al., 2023). Addressing these challenges is essential for enhancing the resilience and sustainability of the potato seed system there by ensuring that farmers have access to high-quality seeds that can improve yields and contribute to national food security. Identifying pathways for improvement in this system is imperative to foster collaboration among stakeholders, enhance regulatory effectiveness, and ultimately support the livelihoods of millions of farmers across the country.

This topic effectively captures the multi-faceted nature of the seed system in Ethiopia, focusing on regulatory challenges, quality control, and potential improvements in the potato seed sector. Furthermore, this comprehensive review paper will provide a well-rounded review of the potato seed system in Ethiopia, highlighting both the challenges and the potential strategies for effective interventions. Therefore, this particular paper seeks to provide insights on potato seed system, regulatory challenges, quality assurance mechanisms ad leverage points for improvement pathways.

## Methods

We have conducted a literature search using different key databases to assess potato seed system, regulatory challenges, seed quality assurance bottlenecks and paths for improvement particular to potato crop in Ethiopia. These included existing reviews and reports using Web of Science, PubMed, Google Scholar, ResearchGate, the CIP database and government annual reports particular to seed system in Ethiopia. Our search employed a variety of terms, including: potato, seed system, seed quality assurance, regulatory framework, seed certification, integrated seed sector development, intervention points for seed sector development.

## Overview of potato seed system in Ethiopia

### Seed system in Ethiopia

The distribution of seeds of improved varieties for farming communities started from the 1950s informally mainly with the establishment of some academic, research and development institutes in Ethiopia. Progressively, the formal seed sector began in the 1970s through several organizational and structural changes and made steady improvement across the seed system ( Alemu et al., 2023). Ethiopia has a pluralistic seed system for crops like potato comprising formal, alternative and informal schemes though scholars put different views on seed system categorization ( Haug et al., 2023; Mulesa et al., 2021; Hirpa et al., 2010). The contribution of informal seed system to planting stock in Ethiopia may be 98.3% with the remaining 1.3% from alternative and the formal sources contributing a very insignificant fraction ( Schulz et al., 2013). Thus, almost all potato farmers in Ethiopia use own-saved seed tubers and seed from their social networks due to various reasons ( Tadesse et al., 2019). As a developing country that lacks a well-functioning seed certification system, Ethiopia adopted the quality declared seed (QDS) for many economically important crops including potato ( Tessema et al., 2023a). QDS, a class below certified seed is guided by standards that are less rigorous but recognized by the regulatory bodies than ordinary seed is permitted in the Ethiopian seed potato system ( Schulz et al., 2013; Tessema et al., 2023a,b). The management and handling of these different seed systems are quite distinct in terms of seed quality, resource utilization, knowledge of seed operating personnel, seed quality assurance/control mechanisms and proscription by law ( Hirpa et al., 2010; Gowda et al., 2017). Furthermore, the productivity of the crop from each seed system/category has considerable difference, for example in Ethiopia; a yield penalty of 35% was reported for some field crops in each hectare when farmers used informal/local seed as their planting material ( Tarekegn and Mogiso, 2020). Thus, sowing/planting quality seed as a source material has considerable yield advantages ( Harahagazwe et al., 2018; Gaur et al., 2020). Furthermore, The primary aim of functional seed systems is to guarantee that seeds are accessible and available to all users, especially smallholder farmers providing an adequate quantity, quality, and variety of seeds to enable sustainable food production and consumption ( Etten et al., 2017). However, in Ethiopia, there is no well delineated seed system that fulfill the required role delegations along the seed value chain of many crops especially VPCs.

The decentralized seed potato system in Ethiopia involves individual farmers, seed grower cooperatives and private seed multipliers at different level of economic, social and agro-ecological settings ( Tadesse et al., 2020; Tessema et al., 2023a; Sulle et al., 2022). However, public seed enterprises are less likely to engage in the potato seed business in the country ( Getnet et al., 2023) and there are no well-organized seed growers except local seed multipliers.

The potato seed system is constrained by tremendous social, economic and biological factors with limited quality assurance capacity ( Tafesse et al., 2020). Most visible, the decentralized seed system has poor monitoring and administration capacity in farmers cooperatives and weak coordination among the seed regulatory authority ( Sulle et al., 2022; Tadesse et al., 2020). The sub-sector is characterized by poor performance, low stakeholder collaboration, weak coordination and inefficient service provision in terms of quality seed production and certification ( Getnet et al. 2023; Tessema et al., 2023b). The mechanisms of applying seed laws, operating procedures and quality standards depends on availability of infrastructure, trained human resources managing the seed system and other interrelated aspects ( Misra et al., 2023). Generally, the seed system in Ethiopia is still at its infant stage to perform key functions of seed systems viz., production and distribution, innovation and regulation along the seed value chain that ultimately affects sustainable potato production and supply ( Tessema et al., 2023a; Hirpa et al., 2010; Etten et al., 2017). This is because the seed sector directly influences three dimensions of food security and nutrition namely; food availability, access and affordability ( Etten et al., 2017).

### Overview of seed regulatory structure in Ethiopia

**Figure 1.**
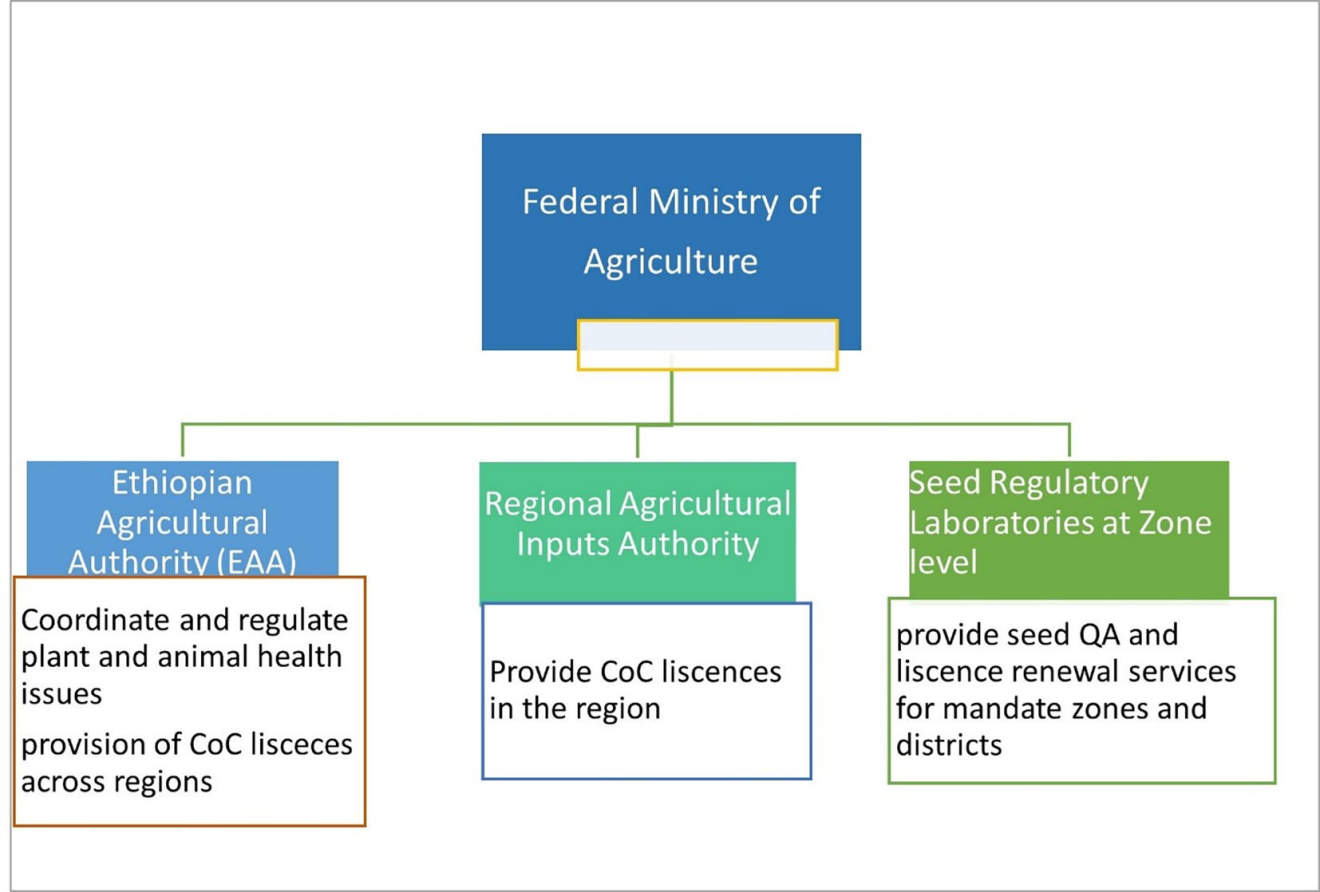
Organizational diagram of seed regulatory authority and their respective roles in Ethiopia.

The seed sector in Ethiopia was coordinated by various state ministers under the Ministry of Agriculture (MoA). Linked to the federal government, the seed quality assurance is cascaded from Federal to Regional regulatory Authorities ( Nigussie et al., 2020). Since 2021, the Ethiopian Agricultural Authority (EAA) under the MoA was established to coordinate all regulatory aspects of the seed sector in Ethiopia Seed Proclamation No. 1262/2021 ( Alemu et al., 2023). Functionally, the seed regulatory laboratories are located at zonal level under the regional agricultural inputs regulatory authority ( Figure 1). Seed regulatory laboratories work mostly on seed of cereal and grain crops and rarely on VPCs that require more advanced pathogen testing facilities and skilled human resource that are currently in short supply in the country ( Tessema et al., 2023a). Seed regulatory laboratories in Ethiopia are essential for testing and certifying seed quality, yet they face significant challenges such as inadequate funding and outdated equipment with limited trained man power ( Tessema et al., 2023b).

The vulnerability of potato to seed-borne diseases and pests requires a strong, efficient and customized regulatory mechanism ( Getnet et al., 2023). The seed regulatory laboratories have a plethora of problems including coordination and capacity bottlenecks to undertake the potato seed quality assurance within the standards set by national or international authorities to achieve the seed sector transformation goal in Ethiopia comparable to cereals and legumes ( Alemu et al., 2023; Nigussie et al., 2020).

### Seed quality assurance and certification standards

Seed quality is a complex trait that involve a combination of plant genetics, seed technology, seed health and molecular biology. It is one of the basic requirements of effective seed industry ( Misra et al., 2023). Some of the classical methods of seed improvement include coating, pelleting, priming, and production of artificial seed ( Wimalasekera, 2015). The seed of VPCs, unlike cereals and pulses are very prone to latent infection by seed and soil borne pathogens such as *Ralstonia solanacearum* and viruses ( Bentley et al., 2018; Tessema et al., 2022).

Seed certification is the process to ensure that the genetic identity and purity of a plant variety are maintained during seed multiplication.

The seed certification schemes rely on standards and procedures implemented at each step of the seed production process. Seeds for sale with a label of “Certified seed”, means that the seed complies with the national quality standards prescribed for certification. Under the seed law, the government seed authority is responsible for the process of seed certification where government carries out the task for seed certification ( Misra et al., 2023). However, in Ethiopia the seed standards set by the national seed regulatory authority are poorly implemented by the seed laboratories ( Tessema et al., 2023a; Nigussie et al., 2020) resulting in farmers planting disease-infected, low quality seed potato. The most detrimental effect of seed-borne pathogens such as potato bacterial wilt (BW) besides affecting immediate yield is the contamination of previously disease-free areas and the spread of new diseases ( Tessema et al., 2023a, b; Singh and Rathaur, 2020).

**Figure 2.**
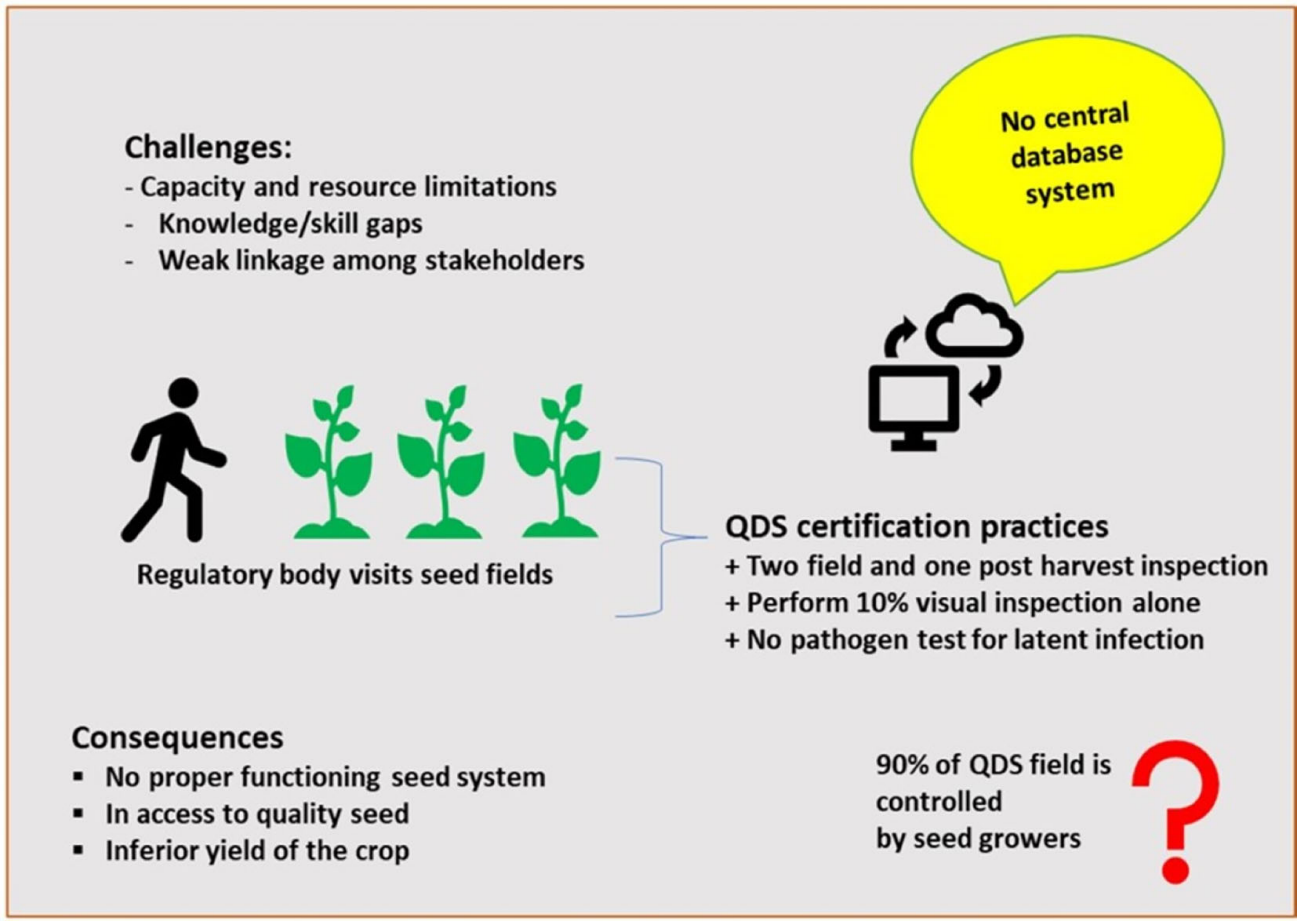
Seed potato inspection and quality assurance practices in Ethiopia.

Without an active seed certification program, the quality of seed tubers is upheld by staff from research organizations and seed potato projects, who work together to maintain minimum quality standards ( Schulz et al., 2013: Getnet et al., 2023). However, as the demand for quality seed increases, this system is starting to hit its production limits. Additionally, its reliance on project interventions makes it unsustainable. Therefore, alternative mechanisms at regional or local levels should be developed, tested, and promoted to ensure that producers of planting materials meet minimum quality standards at smallholders setting. To withstand the seed demand, quality declared seed (QDS) is introduced to the potato seed system that uses slight quality assurance mechanisms ( Tessema et al., 2023a). The QDS system somehow reduces the burden of the seed sector by allowing a 10% external seed inspection by the regulatory body, where as 90% seed quality control is left to the producers ( Figure 2) ( Tessema et al., 2023a). Scholars also recommend various seed regulatory options tailored to the specific circumstances of each region or country, although these recommendations are often poorly implemented ( Gatto et al., 2021).

### Seed regulatory challenges and paths for improvement

Regulatory challenges related to seed of vegetatively propagated crops (VPCs) largely center on varietal identification, certification, and disease control. In contrast to seed-propagated crops, which have clear genetic profiles, VPCs often show considerable variability, making it difficult to establish distinct and consistent varieties ( Gatto et al., 2021). This ambiguity creates hurdles for regulatory agencies responsible for enforcing plant breeding and protection standards ( McEwan et al., 2021; Louwaars et al., 2013) ( Table 1). Moreover, the certification of propagation materials like cuttings and tubers requires thorough testing to ensure they are free from diseases and pests, a process that can be complex and resource-intensive ( Tessema et al., 2023a; Almekinders et al., 2019).

**Table 1.** Seed quality assurance challenges and pathways for improvement.

Aspect	Challenges	Quality assurance issues	Pathways for improvement	Reference
Regulatory framework	Regulatory institutions lack autonomy and role clarity.	Inconsistent seed quality standards across regions.	Streamline and harmonize regulations for efficiency.	Schulz et al., 2013; Tessema et al., 2023b
Quality assurance standards	Seed regulatory agency performs visual inspection without any pathogen test for latent pathogen test.	Most seed testing laboratories do not perform seed potato certification, and those that do rely primarily on visual assessments alone.	Accelerate the approval process and harmonize the seed QA standards.	Tessema et al., 2023b
Access to quality seed tuber	Smallholder farmers often struggle to afford certified seeds, and some of the certified seeds that are available not sufficient to all potato farmers.	Poor traceability of seed quality in the supply chain.	Enhance seed distribution networks and certification.	Haug et al., 2023; Hassena et al., 2023
Training and awareness	Insufficient training for laboratory technicians, inspectors and farmers on seed regulations.	Lack of understanding of quality assurance protocols.	Strengthen the capacity of existing seed labs, regional, and federal regulatory bodies	Almekinders et al., 2019
Research and development	Inadequate funding for seed sector development.	Limited innovation in developing disease-resistant varieties and appropriate disease detection procedures.	Increase investment in seed research and development.	Schulz et al., 2013
Logistics and laboratory facilities	Logistical and administration capacity of seed regulatory laboratories are limited.	Most of seed regulatory laboratories could not oversee their mandate areas due to capacity limitation.	Reinforce the seed regulatory laboratories to perform quick and standard quality assurance activity.	McEwan et al., 2023; Hassena et al., 2023

Additionally, the intellectual property framework for vegetatively propagated crops introduces further regulatory complications ( Gatto et al., 2021). Current plant variety protection laws may not sufficiently accommodate the specific traits of these crops, potentially hindering the ability to secure patents or protection certificates. Traceability is another critical issue, as it is essential to track the origins and quality of vegetative materials throughout the supply chain to uphold biosecurity and market integrity ( Zheng et al., 2023). As nations implement varying regulations for the import and export of these crops, navigating the international regulatory landscape becomes more challenging, highlighting the need for collaboration among stakeholders to establish effective and coherent regulatory systems ( McEwan et al., 2021; Gatto et al., 2021).

In Ethiopia, research conducted in collaboration with the Ethiopian Institute of Agricultural Research (EIAR) and the regional Bureaus of Agriculture and Natural Resource Development revealed that six major potato viruses and bacterial wilt are prevalent throughout all levels of the seed system (Sulle e al., 2022; Tessema et al., 2023b, 2024; McEwan et al., 2023). However, an in-depth assessment of seed testing laboratories and seed inspection processes indicated limited resources and capabilities for implementing seed quality assurance.

One of the most significant challenges in potato seed systems is the infiltration of counterfeit or substandard seeds into the supply chain ( Hirpa et al., 2010; Sulle et al., 2022). The lack of a robust, digitized, and automated system makes it relatively easy for these fraudulent seeds to enter the market (Tessema personal communication). This not only undermines seed quality but also leads to substantial losses for farmers who unknowingly buy and plant these counterfeit seeds ( Tessema et al., 2023a; Sulle et al., 2022). Additionally, the widespread presence of such seeds compromises the integrity of the seed industry and negatively impacts overall crop production and harvest quality ( Buddenhagen et al., 2017; Tessema et al., 2024). The absence of digitization and automation in the seed delivery system contributes to seeds’ need for visibility and traceability (Tessema personal communication) ( Figure 2).

This lack of transparency poses risks related to quality, expiry, counterfeiting, and accountability in the seed supply chain since the seed quality is often unobservable through visual inspection by the farmers ( McEwan et al., 2021).

Improving seed quality assurance is vital for boosting agricultural productivity and sustainability ( Wimalasekera, 2015). Seed potatoes are fundamental to both the quality and yield of potato crops, acting as the first link in the entire potato value chain ( Yves and Quere, 2023). Here are some key points to consider for improving seed systems:

• Working together to harmonize seed regulations and ensure better compliance through identification of intervention points for improved seed system development.
• Engaging farmers, researchers, policy makers and the public in the regulatory process is imperative to build trust among value chain actors and lead to better outcomes.
• Maximizing thew potential of the informal seed sector, since the majority of the seed smallholder farmers used is sourced from informal see system.
• Improving public understanding of seed technologies and regulatory practices can counter misinformation and encourage acceptance.
• Integrating sustainability criteria into seed regulations can promote the development of environmentally friendly practices that in turn builds the capacity of smallholder seed producers.

## Conclusions

Ethiopia’s potato seed system faces significant regulatory challenges that hinder its development and efficiency. One of the primary issues is the lack of a coherent regulatory framework that governs seed production and distribution. Existing regulations are often outdated or poorly enforced, leading to inconsistencies in quality and availability. Furthermore, the bureaucratic processes involved in obtaining seed certification can be cumbersome and time-consuming, discouraging local farmers and seed producers from engaging in formal markets. The interplay between various regulatory bodies can create confusion, resulting in overlapping mandates and insufficient support for stakeholders at different levels. Addressing these regulatory challenges is crucial for creating a more robust potato seed system that can better meet the needs of Ethiopian farmers.

To enhance Ethiopia’s potato seed system, a multifaceted approach is necessary, focusing on strengthening regulatory frameworks, improving quality assurance, and fostering collaboration among stakeholders. First, reforming and streamlining the regulatory processes can help reduce bureaucratic bottlenecks, making it easier for seed producers to operate and access markets. Second, investing in research and development can lead to the creation of more resilient and high-yielding potato varieties that are better suited to local conditions. Engaging farmers in participatory breeding programs can also empower them to influence seed variety development. Lastly, promoting public-private partnerships can facilitate knowledge transfer and resource sharing, ultimately leading to a more integrated and effective potato seed system. By addressing these key areas, Ethiopia can significantly improve its potato seed system, benefiting farmers and enhancing food security in the country as well.

## Additional information

### Author contribution

• Lemma Tessema conceived and designed the experiments, performed the experiments, prepared figures and/or tables, authored or reviewed drafts of the article, and approved the final draft.
• Ebrahim Seid performed the experiments, authored or reviewed drafts of the article, and approved the final draft.

## Data Availability

No data are associated with this article.

## References

[ref1] Alemu D Bishaw Z Zeray T : National Seed Sector Coordination in Ethiopia: Status, Challenges, and Way Forward. 2023. Reference Source

[ref2] Almekinders CJM Walsh S Jacobsen KS : Why interventions in the seed systems of roots, tubers and bananas crops do not reach their full potential. *Food Secur.* 2019;11:23–42. 10.1007/s12571-018-0874-4

[ref3] Bentley JW Andrade-Piedra J Demo P : Understanding root, tuber, and banana seed systems and coordination breakdown: A multi-stakeholder framework. *J. Crop Improv.* 2018;32(5):599–621. 10.1080/15427528.2018.1476998

[ref4] Buddenhagen CE Hernandez Nopsa JF Andersen KF : Epidemic Network Analysis for Mitigation of Invasive Pathogens in Seed Systems: Potato in Ecuador. *Phytopathology.* 2017;107(10):1209–1218. 10.1094/PHYTO-03-17-0108-FI 28742457

[ref5] Devaux A Goffart JP Kromann P : The Potato of the Future: Opportunities and Challenges in Sustainable Agri-food Systems. *Potato Res.* 2021;64:681–720. 10.1007/s11540-021-09501-4 34334803 PMC8302968

[ref6] Etten JV López Noriega I Fadda C : The contribution of seed systems to crop and tree diversity in sustainable food systems. 2017. [Accessed on 15 April 2024]. Reference Source

[ref7] Gatto M Le PD Pacillo G : Policy options for advancing seed systems for vegetatively propagated crops in Vietnam. *J. Crop Improv.* 2021;35(6):763–789. 10.1080/15427528.2021.1881011

[ref8] Gaur A Kumar A Kiran R : Importance of Seed-Borne Diseases of Agricultural Crops: Economic Losses and Impact on Society. Kumar R Gupta A , editors. *Seed-Borne Diseases of Agricultural Crops: Detection, Diagnosis & Management.* Singapore: Springer;2020. 10.1007/978-981-32-9046-4_1

[ref9] Getnet M Teshome A Snel H : Potato seed system in Ethiopia: challenges, opportunities, and leverage points. *Stichting Wageningen Research Ethiopia, Addis Ababa.* 2023. SWRE-RAISE-FS-23-025. 10.18174/649748

[ref10] Gowda M Worku M Nair SK : *Quality Assurance/Quality Control (QA/QC) in Maize Breeding and Seed Production: Theory and Practice.* Nairobi: CIMMYT;2017. 978-9966-1971-9-1.

[ref11] Harahagazwe D Condori B Barreda C : How big is the potato (Solanum tuberosum L.) yield gap in Sub-Saharan Africa and why? A participatory approach. *Open Agric.* 2018;3:180–189. 10.1515/opag-20180019

[ref12] Hassena M Alemu D Dey B : Seed policy provisions and operational challenges in Ethiopia. A Feed the Future Global Supporting Seed Systems for Development activity (S34D) report. 2023. Reference Source

[ref13] Haug R Hella JP Mulesa TH : Seed systems development to navigate multiple expectations in Ethiopia, Malawi and Tanzania. *World Dev. Sustain.* 2023;3:100092. 10.1016/j.wds.2023.100092

[ref14] Hirpa A Meuwissen MPM Tesfaye A : Analysis of Seed Potato Systems in Ethiopia. *Am. J. Potato Res.* 2010;87:537–552. 10.1007/s12230-010-9164-1

[ref15] Jennings SA Koehler A-K Nicklin KJ : Global Potato Yields Increase Under Climate Change with Adaptation and CO2 Fertilisation. *Front. Sustain. Food Syst.* 2020;4:519324. 10.3389/fsufs.2020.519324

[ref16] Katrin A Tara F Indulekha T : Laws and regulations enabling and restricting Africa’s vegetable seed sector. *Int. J. Agric. Sustain.* 2023;21(1):2210005. 10.1080/14735903.2023.2210005

[ref17] Louwaars NP Boef WS de Edeme J : Integrated Seed Sector Development in Africa: A Basis for Seed Policy and Law. *J. Crop Improv.* 2013;27(2):186–214. 10.1080/15427528.2012.751472

[ref18] McEwan MA Almekinders CJ Andrade-Piedra JJ : “Breaking through the 40% adoption ceiling: Mind the seed system gaps.” A perspective on seed systems research for development in One CGIAR. *Outlook Agric.* 2021;50(1):5–12. 10.1177/0030727021989346 33867584 PMC8022077

[ref19] McEwan MA Kumar L Legg J : Enhancing seed quality assurance: Options for vegetatively propagated crops. ISSD Africa Topical Synthesis Paper. 2023. [Accessed on 23 April 2024]. Reference Source

[ref20] Mijena GM Gedebo A Beshir HM : Ensuring food security of smallholder farmers through improving productivity and nutrition of potato. *J. Agric. Food Res.* 2022;10:100400. 10.1016/j.jafr.2022.100400

[ref21] Misra MK Harries A Dadlani M : Role of Seed Certification in Quality Assurance. Dadlani M Yadava DK , editors. *Seed Science and Technology.* Singapore: Springer;2023. 10.1007/978-981-19-5888-5_12

[ref22] Mulesa TH Dalle SP Makate C : Pluralistic seed system development: a path to seed security? *Agronomy.* 2021;11(2):372. 10.3390/agronomy11020372

[ref23] Nigussie M Kalsa K Ayana A : Status of Seed Quality Control and Assurance in Ethiopia: Required Measures for Improved Performance. *Technical report.* 2020. 10.13140/RG.2.2.34415.87202

[ref24] Schulz S Woldegiorgis G Hailariam G : Sustainable seed potato production in Ethiopia: from farm-saved to quality declared seed. Woldegiorgis G Schulz S Berihun B , editors. *Proceedings of the National Workshop on Seed Potato Tuber Production and Dissemination: Experiences, Challenges and Prospects, 12-14 March 2012.* Bahir Dar, Ethiopia: Ethiopian Institute of Agricultural Research and Amhara Regional Agricultural Research Institute;2013; pp.60–80. Reference Source

[ref25] Singh D Rathaur PS : Detection of Seed and Propagating Material-Borne Bacterial Diseases of Economically Important Crops. Kumar R Gupta A , editors. *Seed-Borne Diseases of Agricultural Crops: Detection, Diagnosis & Management.* Singapore: Springer;2020. 10.1007/978-981-32-9046-4_6

[ref26] Sulle E Pointer R Kumar L : *Inventory of novel approaches to seed quality assurance mechanisms for vegetatively propagated crops (VPCs) in seven African countries.* International Institute of Tropical Agricultu re (IITA). International Potato Center (CIP);2022;48. 10.4160/9789290606543

[ref27] Tadesse Y Almekinders CJM Griffin D : Collective Production and Marketing of Quality Potato Seed: Experiences from Two Cooperatives in Chencha, Ethiopia. *Forum Dev. Stud.* 2020;47(1):139–156. 10.1080/08039410.2019.1635523

[ref28] Tadesse Y Almekinders CJM Schulte RPO : Potatoes and livelihoods in Chencha, southern Ethiopia. *NJAS Wageningen J. Life Sci.* 2019;88:105–111. 10.1016/j.njas.2018.05.005

[ref29] Tafesse S Lie R Mierlo B van : Analysis of a monitoring system for bacterial wilt management by seed potato cooperatives in Ethiopia: Challenges and future direction. *Sustainability.* 2020;12:3580. 10.3390/su12093580

[ref30] Tarekegn K Mogiso M : Assessment of improved crop seed utilization status in selected districts of Southwestern Ethiopia. *Cogent Food & Agriculture.* 2020;6(1):1816252. 10.1080/23311932.2020.1816252

[ref31] Tessema L Kakuhenzire R McEwan M : *Latent bacterial wilt and viral infection burden in the seed potato system in Ethiopia: Policy implications for seed potato. Policy Brief 02.* International Potato Center;2023b. Reference Source

[ref32] Tessema L Kakuhenzire R Seid E : Detection of six potato viruses using double antibody sandwich ELISA from in vitro, screen house and field grown potato crops in Ethiopia. *Discov. Appl. Sci.* 2024;6:79. 10.1007/s42452-023-05619-x

[ref33] Tessema L Negash W Kakuhenzire R : Seed health trade-offs in adopting quality declared seed in potato farming systems. *Crop Sci.* 2023a;1–9. 10.1002/csc2.21025

[ref34] Tessema L Seid E W/Giorgis G : Incidence and occurrence of latent *Ralstonia solanacearum* infection in seed potato from farmer seed grower cooperatives in Southern and Central Ethiopia. *Potato Res.* 2022;65:649–662. 10.1007/s11540-022-09541-4

[ref35] Wijesinha-Bettoni R Mouille B : The Contribution of Potatoes to Global Food Security, Nutrition and Healthy Diets. *Am. J. Potato Res.* 2019;96:139–149. 10.1007/s12230-018-09697-1

[ref36] Wimalasekera R : Role of Seed Quality in Improving Crop Yields. Hakeem K , editor. *Crop Production and Global Environmental Issues.* Cham: Springer;2015. 10.1007/978-3-319-23162-4_6

[ref37] Yves LH Quere B : Seed potato production, certification, and trade. Caliskan ME Bakhsh A Jabran K , editors. *Potato Production Worldwide.* Academic Press;2023; pp.241–260. 10.1016/B978-0-12-822925-5.00014-1

[ref38] Zheng Y Xu Y Qiu Z : Blockchain Traceability Adoption in Agricultural Supply Chain Coordination: An Evolutionary Game Analysis. *Agriculture.* 2023;13:184. 10.3390/agriculture13010184

