# Supplementary material for: Ethiopia's Potato Seed System: Regulatory Challenges, Quality Assurance Issues, and Pathways for Improvement ─ A mini review
Source: F1000Res. 2025 Feb 10;13:1348. Originally published 2024 Nov 11. [Version 2] doi: 10.12688/f1000research.158093.2 (PMC11782929; doi:10.12688/f1000research.158093.2)
Supplement: Peer review history

# Reviewer report: Reviewer response for version 1

**DOI:** 10.5256/f1000research.173639.r357063

**Published:** 2025-01-30

**Authors:** Femi Emmanuel Awosanmi

**Affiliations:** 1 OAU · Seed Science Unit, Department of Crop Production and Protection, Faculty of Agriculture, Obafemi Awolowo University |, Osun, Nigeria

The Review article gave insight into the Ethiopian Seed System and highlighted the challenges facing the sector and the consequences of these on the Potato production industry in particular. The authors also made good, significant suggestions to tackle these challenges to establish a viable potato seed production system that would enhance the yield and productivity of potato in Ethiopia. 

 However, it will be appropriate for the authors to maintain a definite pattern of citing references throughout the article; provide the full meaning of VPC where it was mentioned; give an explanation on COC licences because of audience who are not based or familiar with Ethiopia; and reflect the National Seed Regulatory authority mentioned on the Organizational diagram as mentioned in the text. 

 Also, the conclusion that "The interplay between various regulatory bodies can create confusion, resulting in overlapping mandates and insufficient support for stakeholders at different levels" was not clearly reflected in the main text.

Is the review written in accessible language?

Yes

Are all factual statements correct and adequately supported by citations?

Yes

Are the conclusions drawn appropriate in the context of the current research literature?

Partly

Is the topic of the review discussed comprehensively in the context of the current literature?

Yes

Reviewer Expertise:

Seed Science and Technology

I confirm that I have read this submission and believe that I have an appropriate level of expertise to confirm that it is of an acceptable scientific standard.

# Reviewer report: Reviewer response for version 1

**DOI:** 10.5256/f1000research.173639.r340501

**Published:** 2025-01-15

**Authors:** Kwame Ogero

**Affiliations:** 1 International Potato Centre, Mwanza, Tanzania

The review gives a good summary of the bottlenecks facing the potato seed systems in Ethiopia. The regulatory issues and quality assurace practices are well captured and a clear way forward presented. I recommend the article for indexing  after an editorial review to correct typos and structure of some sentences.

Is the review written in accessible language?

Yes

Are all factual statements correct and adequately supported by citations?

Yes

Are the conclusions drawn appropriate in the context of the current research literature?

Yes

Is the topic of the review discussed comprehensively in the context of the current literature?

Yes

Reviewer Expertise:

Seed systems, pest and disease management

I confirm that I have read this submission and believe that I have an appropriate level of expertise to confirm that it is of an acceptable scientific standard.
